# Genomic sequence of yellow fever virus from a Dutch traveller returning from the Gambia-Senegal region, the Netherlands, November 2018

**DOI:** 10.2807/1560-7917.ES.2019.24.4.1800684

**Published:** 2019-01-24

**Authors:** My VT Phan, Sarwa Darwish Murad, Annemiek A van der Eijk, Herold J. Metselaar, Hermien Hartog, Femme Harinck, Corine H GeurtsvanKessel, Richard Molenkamp, Matthew Cotten, Marion PG Koopmans

**Affiliations:** 1 Department of Viroscience, Erasmus MC, Rotterdam, the Netherlands; 2 Department of Gastroenterology and Hepatology, Erasmus MC, Rotterdam, the Netherlands; 3 Division of Hepato-Pancreato-Biliary and Transplant Surgery, Department of Surgery, Erasmus MC, Rotterdam, the Netherlands

**Keywords:** yellow fever, yellow fever virus, YFV, return traveller, Gambia-Senegal, liver failure, rapid outbreak sequencing, outbreak

## Abstract

In November 2018, yellow fever was diagnosed in a Dutch traveller returning from a bicycle tour in the Gambia-Senegal region. A complete genome sequence of yellow fever virus (YFV) from the case was generated and clustered phylogenetically with YFV from the Gambia and Senegal, ruling out importation into the Netherlands from recent outbreaks in Brazil or Angola. We emphasise the need for increased public awareness of YFV vaccination before travelling to endemic countries.

We report the genomic sequence of yellow fever virus (YFV) genome directly from clinical samples from an unvaccinated Dutch traveller returning from the Gambia-Senegal region, where yellow fever (YF) is endemic. This report sends a reminder of the importance of vaccination for travellers to endemic areas and furthermore shares with the community a YFV genome sequence identified from an area with limited YFV sequence coverage.

## Case description

The case was a healthy, unvaccinated adult in his mid-20s who had travelled to the Gambia and Senegal for a 17-day bicycle tour in November 2018 (14 days in the Gambia and 3 days in Senegal). The patient had reported insect bites while travelling. During the returning flight to the Netherlands on 17 November, the patient developed fever and chills and then quickly progressed to acute kidney injury and fulminant liver failure for which he was hospitalised 20 November [1]. On 21 November, the patient was referred to the Erasmus Medical Centre (Rotterdam, the Netherlands) for treatment. Based on the clinical presentation and the recent travel history, YF was suspected and confirmed by a real-time PCR diagnostic assay on samples collected on 19 November and confirmed again on samples collected on 21 November. The patient was discharged 3 weeks after admission and has fully recovered from the infection. Full details of the clinical course and the advanced treatment will be described elsewhere.

## Sample processing and agnostic deep sequencing

An in-house standard PCR for YFV yielded a Ct-value of 14 for a plasma sample collected on 19 November. This sample was prepared for whole genome sequencing as follows. Total nucleic acid was extracted using Roche MagNa Pure high performance extraction kit (Roche, Mannheim, Germany), followed by reverse transcription using random hexamer primers that avoid rRNA binding. Second strand synthesis was performed as previously described [2], followed by standard Ion Torrent library preparation as per manufacturer’s instruction. Deep sequencing was performed on the S5-XL sequencer, generating ca 10 million short reads of median length 263 nt. Short and low quality reads (< 75 nt, Phred score < 25) were removed and the remaining reads were de novo assembled to larger contigs using SPAdes v.3.13.0 [3]. The YFV sequence contigs were identified using Usearch [4] against a set of viral family protein databases. A complete YFV genome (10771 nt) was obtained from the analysis.

## Alignment and phylogenetic analysis

This YFV genome (GenBank accession number MK292067) and all available YFV genomes retrieved from GenBank (n=188) were aligned using MUSCLE [5], manually checked in AliView [6], and trimmed to the complete Open Reading Frame (ORF). The evolutionary model testing was implemented in IQ-TREE [7] using the Akaike Information Criterion (AIC).

A maximum-likelihood phylogenetic tree was constructed using the sequence alignment in RAxML [8] under the GTR + Γ_4_ model of evolution, which was determined as the best-fitted model, bootstrapped with 100 pseudoreplicates. The resulting tree was visualised and edited in FigTree v1.4.2 (http://tree.bio.ed.ac.uk/software/figtree/) and mid-point rooted for clarity.

## Clustering with other YFV sequences

The reported YFV genome was found to belong to the West Africa genotype according to a genotyping tool (http://krisp.ukzn.ac.za/app/typingtool/yellowfevervirus/) and in a maximum-likelihood phylogenetic tree (Figure). The reported genome was most closely related to a Gambian YFV genome from 2001 [9] with 98.3% nt identity across the entire genome and 195 nt differences and to Senegalese YFV genomes identified in 2000 [10]. Earlier Senegalese YFV genomes from 1995, 1996, 2001 and 2005 belonged to related but distinct lineages within the West Africa genotype.

**Figure:**
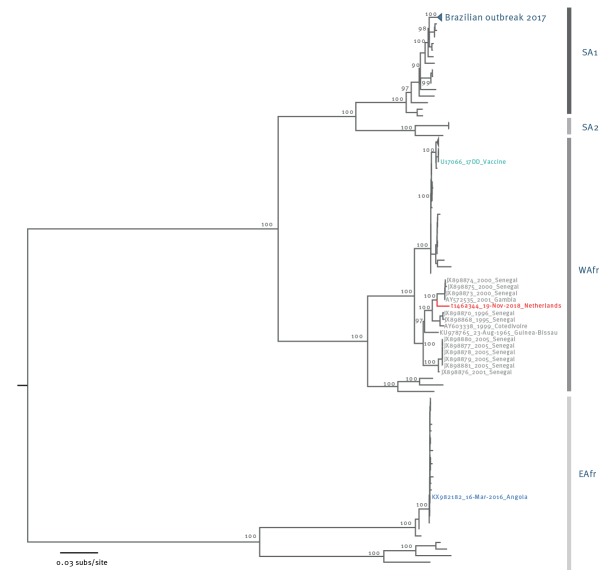
Maximum-likelihood phylogenetic tree of the complete yellow fever virus genomes including sequence from Dutch traveller to the Gambia and Senegal in November 2018

EAfr: East Africa genotype; SA1: South American I genotype; SA2: South American II genotype; WAfr: West Africa genotype; YVF: yellow fever virus.

Brazilian outbreak clade was indicated in dark blue (triangle). The reported YFV genome is indicated in red; the YFV vaccine strain is indicated in turquoise and the YFV strain from Angola outbreak is indicated in blue.

The genotypes of each YFV clade (as defined in [11]) are shown and abbreviated. The phylogeny was mid-point rooted for clarity and only bootstrap values for major clades were shown. The scale bar is shown in units of number of nt substitutions per site (subs/site).

The viral sequence from the patient was clearly distinct from viral sequences from the recent large outbreaks in Brazil (SA1 lineage, Figure [11]) and Angola (EAfr lineage, Figure [12]), suggesting that the YFV infection was likely a sylvatic case derived from locally circulating viruses in the Gambia and Senegal and not a new introduction of the virus into this region. However, it should be noted that there is a paucity of publicly available YFV genome sequences from Africa.

## Discussion and conclusion

YF is a severe, mosquito-borne flavivirus infection caused by YFV, that is estimated to result in 78,000 deaths annually in Africa alone [13,14]. YFV transmission continues in tropical regions of the world with larger recent outbreaks reported in Brazil [11] and Angola. A smaller number of cases are reported from additional countries as listed on World Health Organization (WHO) news on disease outbreak [15]. Given the identification of co-circulating YFV lineages in regions over several years [9,11,15-22] and the general lack of sampling in the animal reservoir, it is plausible that more diversity may be observed with more comprehensive sequencing of newly diagnosed cases. Such surveillance in this part of the world would provide further knowledge and understanding of YFV transmission and evolution, which would be valuable in supporting the YF epidemic elimination initiative.

Although an effective and safe vaccine has been available since 1939 [23], vaccine coverage is still insufficient and a limited vaccine supply coupled with human population increases has led to high numbers of unvaccinated people living in endemic regions [24]. There have been several reports of YF cases in unvaccinated travellers returning from endemic regions in the past years such as to Belgium from the Gambia [9,16,18], to China from Angola [17,19] and to the Netherlands from Suriname [20,21] or from Brazil [22]. Furthermore, returning travellers may serve as sentinels for local outbreaks of pathogenic viruses that may have not yet been documented or adequately reported.

The WHO has launched a programme to eliminate YF epidemics in regions at risk for cases from enzootic circulation or new introductions [25]. A key component of a successful elimination campaign is the ability to detect new cases and to understand the ecology of YF in regions at risk. Whole genome viral sequences can provide important data for tracking viruses within and between outbreaks [11,26-28]. Having a rapid whole-genome confirmation of a YFV infection and placing the sequence in the context of the global YFV phylogenetics is crucial for ruling out alternate transmission possibilities such as importation and introduction of YFV into the Netherlands from the recent large YFV outbreaks in Brazil or Angola. This work also highlights the need to remain alert for unexpected infectious disease aetiologies in returning travellers and the need to consider vaccination before travelling to regions where YFV is endemic, even if the vaccination is not required by border control agencies or when there are no reports of human cases of YF in these regions.

## Data availability

The YFV genomic sequence reported here is available on GenBank with the accession number MK292067.

## References

[1] ProMED-mail PRO/AH/EDR> Yellow fever - Africa (22): Nigeria (ED), Netherlands ex Gambia/Senegal. Archive number 20181123.6160612. Available from: http://www.promedmail.org

[2] Phan MVT Anh PH Cuong NV Munnink BBO van der Hoek L My PT Unbiased whole-genome deep sequencing of human and porcine stool samples reveals circulation of multiple groups of rotaviruses and a putative zoonotic infection. Virus Evol. 2016;2(2):vew027. 10.1093/ve/vew027 28748110 PMC5522372

[3] Bankevich A Nurk S Antipov D Gurevich AA Dvorkin M Kulikov AS SPAdes: a new genome assembly algorithm and its applications to single-cell sequencing. J Comput Biol. 2012;19(5):455-77. 10.1089/cmb.2012.0021 22506599 PMC3342519

[4] Edgar RC Search and clustering orders of magnitude faster than BLAST. Bioinformatics. 2010;26(19):2460-1. 10.1093/bioinformatics/btq461 20709691

[5] Edgar RC MUSCLE: multiple sequence alignment with high accuracy and high throughput. Nucleic Acids Res. 2004;32(5):1792-7. 10.1093/nar/gkh340 15034147 PMC390337

[6] Larsson A AliView: a fast and lightweight alignment viewer and editor for large datasets. Bioinformatics. 2014;30(22):3276-8. 10.1093/bioinformatics/btu531 25095880 PMC4221126

[7] Nguyen L-T Schmidt HA von Haeseler A Minh BQ IQ-TREE: a fast and effective stochastic algorithm for estimating maximum-likelihood phylogenies. Mol Biol Evol. 2015;32(1):268-74. 10.1093/molbev/msu300 25371430 PMC4271533

[8] Stamatakis A RAxML-VI-HPC: maximum likelihood-based phylogenetic analyses with thousands of taxa and mixed models. Bioinformatics. 2006;22(21):2688-90. 10.1093/bioinformatics/btl446 16928733

[9] Bae HG Drosten C Emmerich P Colebunders R Hantson P Pest S Analysis of two imported cases of yellow fever infection from Ivory Coast and The Gambia to Germany and Belgium. J Clin Virol. 2005;33(4):274-80. 10.1016/j.jcv.2004.12.001 16036176

[10] Stock NK Laraway H Faye O Diallo M Niedrig M Sall AA Biological and phylogenetic characteristics of yellow fever virus lineages from West Africa. J Virol. 2013;87(5):2895-907. 10.1128/JVI.01116-12 23269797 PMC3571399

[11] Faria NR Kraemer MUG Hill SC Goes de Jesus J Aguiar RS Iani FCM Genomic and epidemiological monitoring of yellow fever virus transmission potential. Science. 2018;361(6405):894-9. 10.1126/science.aat7115 30139911 PMC6874500

[12] Simon-Loriere E Faye O Prot M Casademont I Fall G Fernandez-Garcia MD Autochthonous Japanese Encephalitis with Yellow Fever Coinfection in Africa. N Engl J Med. 2017;376(15):1483-5. 10.1056/NEJMc1701600 28402771

[13] Garske T Van Kerkhove MD Yactayo S Ronveaux O Lewis RF Staples JE Yellow Fever in Africa: estimating the burden of disease and impact of mass vaccination from outbreak and serological data. PLoS Med. 2014;11(5):e1001638. 10.1371/journal.pmed.1001638 24800812 PMC4011853

[14] Paules CI Fauci AS Yellow Fever - Once Again on the Radar Screen in the Americas. N Engl J Med. 2017;376(15):1397-9. 10.1056/NEJMp1702172 28273000

[15] World Health Organization (WHO). Emergencies preparedness, response. Yellow fever. Geneva: WHO; 2019. Available from: https://www.who.int/csr/don/archive/disease/yellow_fever/en/

[16] Colebunders R Mariage J-L Coche J-Ch Pirenne B Kempinaire S Hantson P A Belgian traveler who acquired yellow fever in the Gambia. Clin Infect Dis. 2002;35(10):e113-6. 10.1086/344180 12410495

[17] Song R Guan S Lee SS Chen Z Chen C Han L Late or lack of vaccination linked to importation of yellow fever from angola to China. Emerg Infect Dis. 2018;24(7):1383-6. 10.3201/eid2407.171868 29723485 PMC6038747

[18] World Health Organization (WHO). Emergencies preparedness, response. 2001 - Imported case of yellow fever in Belgium – Update. Geneva: WHO; 2011. Available from: http://www.who.int/csr/don/2001_11_15/en/

[19] World Health Organization (WHO). Emergencies preparedness, response. Yellow fever-China. Geneva: WHO; 2016. Available from: http://www.who.int/csr/don/22-april-2016-yellow-fever-china/en/

[20] World Health Organization (WHO). Emergencies preparedness, response. 2000 - Imported case of yellow fever in the Netherlands. Geneva: WHO; 2000. Available from: http://www.who.int/csr/don/2000_02_25/en/

[21] Virological. Yellow Fever Virus genomic sequence from a Dutch traveller returning from Suriname. 2017. Available from: http://virological.org/t/yellow-fever-virus-genomic-sequence-from-a-dutch-traveller-returning-from-suriname/185

[22] World Health Organization (WHO). Emergencies preparedness, response. Yellow fever-Brazil. Geneva: WHO; 2018. Available from: http://www.who.int/csr/don/22-january-2018-yellow-fever-brazil/en/

[23] Frierson JG The yellow fever vaccine: a history. Yale J Biol Med. 2010;83(2):77-85. 20589188 PMC2892770

[24] Shearer FM Longbottom J Browne AJ Pigott DM Brady OJ Kraemer MUG Existing and potential infection risk zones of yellow fever worldwide: a modelling analysis. Lancet Glob Health. 2018;6(3):e270-8. 10.1016/S2214-109X(18)30024-X 29398634 PMC5809716

[25] World Health Organization (WHO). Emergencies preparedness, response. Eliminating Yellow Fever Epidemics (EYE) Strategy: Meeting demand for yellow fever vaccines. Geneva: WHO; 2018. Available from: https://www.who.int/csr/disease/yellowfev/meeting-demand-for-vaccines/en/

[26] Dudas G Carvalho LM Bedford T Tatem AJ Baele G Faria NR Virus genomes reveal factors that spread and sustained the Ebola epidemic. Nature. 2017;544(7650):309-15. 10.1038/nature22040 28405027 PMC5712493

[27] Arias A Watson SJ Asogun D Tobin EA Lu J Phan MVT Rapid outbreak sequencing of Ebola virus in Sierra Leone identifies transmission chains linked to sporadic cases. Virus Evol. 2016;2(1):vew016. 10.1093/ve/vew016 28694998 PMC5499387

[28] Cotten M Watson SJ Kellam P Al-Rabeeah AA Makhdoom HQ Assiri A Transmission and evolution of the Middle East respiratory syndrome coronavirus in Saudi Arabia: a descriptive genomic study. Lancet. 2013;382(9909):1993-2002. 10.1016/S0140-6736(13)61887-5 24055451 PMC3898949

